# Detection of *Hepatozoon* spp. in dogs in Shiraz, southern Iran and its effects on the hematological alterations 

**DOI:** 10.22099/mbrc.2023.47151.1821

**Published:** 2023

**Authors:** Ehsan Zoaktafi, Hassan Sharifiyazdi, Nooshin Derakhshandeh, Farnoosh Bakhshaei-Shahrbabaki

**Affiliations:** Department of Clinical Sciences, School of Veterinary Medicine, Shiraz University, Shiraz, Iran

**Keywords:** Hepatozoon canis, Molecular detection, Dog, Hematological analysis

## Abstract

Canine hepatozoonosis is a tick-transmitted apicomplexan infection caused by two species of Hepatozoon, *H. canis*, and *H. americanum*. The present research aimed at detection of Hepatozoon spp. in dogs and its effects on hematological alterations. Blood samples were taken from 108 dogs to assess Hepatozoon spp. Phylogenetic analysis was performed based on the 18S rDNA marker by PCR assay and Giemsa-stained blood smear examination. Of the 108 blood samples of dogs tested in the present study, eight (7.40%, 95% CI: 3.25-14.07%) were positive by the Hepatozoon-specific PCR assay. However, in the microscopic examination, only one sample (0.93%) was positive. All of the sequenced samples were H. canis. The Hepatozoon sequences obtained from PCR amplicons in the canine-positive cases exhibited 100% similarity to each other and 98.47-100% similarity to other relevant sequences in GenBank. These findings represent the first molecular evidence of *H. canis* in dog populations in South Iran. Furthermore, according to the hematological analysis, significantly higher average numbers of neutrophils and lymphocytes were found in the infected group compared to the non-infected dogs. In this study, no statistically significant connection (P<0.05) was observed between *H. canis* infection and the examined risk factors.

## INTRODUCTION

Hepatozoon spp. is the tick-borne apicomplexan parasite mainly found in mammalian neutrophils and monocytes. About 50 species can infect mammals. To date, Hepatozoon americanum and Hepatozoon canis have been recognized as canine hepatozoonosis causes [1]. The previous studies have confirmed that canine hepatic zoonosis diseases can be transmitted by ticks, transplacental transmission from infected maternal animals to fetuses, and by ingestion of prey species such as *H. americanum* paratenic hosts. Similarly, ticks have been identified as the main route of transmission, especially Rhipicephalus sanguineus and Amblyomma maculatum [2]. 

The prevalence of *H. americanum* and *H. canis* is common in tropical and subtropical regions, as well as the US southeastern regions [3]. The prevalence of canine hepatozoonosis in Iran was first reported in Mashhad [4]. 

Much of the severity in pathogenesis stems from the immune system, concomitant infections, Leishmaniosis, Ehrlichiosis, and Babesiosis. Six clinical indications vary from asymptomatic to life-threatening conditions depending on the infection severity. While *H. canis* is more common in lymph nodes and may induce mild lethargy and anemia, H. americanum is more common in skeletal muscle and may cause severe lameness and myositis [5]. 

The most common *H. canis* diagnostic method is a Giemsa-stained blood smear microscopic examination. Based on previous studies, buffy-coat smears are more sensitive than blood smears in spotting the gamonts in the neutrophils and monocytes' cytoplasm. The limitation of circulating parasitemia causes a notable paucity of *H. americanum* gomonts in blood smears [6]. The number of organisms detected in blood smears depends on the disease's severity. One of the challenges faced by blood smear microscopic examination is the asymptomatic carriers. The serological test, the enzyme-linked immunosorbent assay (ELISA), and the indirect fluorescent antibody test (IFAT) are used for epidemiological studies. PCR has been used as a sensitive and specific method to detect Hemoprotozoan DNA in infected dogs' peripheral blood. However, it may offer reliable information on the global prevalence of hepatozoonosis. Criado-Fornelio et al. (2003) figured out that DNA detection of H. canis is mainly based on PCR assay that targets the nuclear 18S ribosomal (r) RNA marker [7].

The present research appears to be the first to evaluate the prevalence of canine liver zoonotic diseases in southern Iran. The aim of this molecular and phylogenetic study was to identify the causative agent(s) of canine hepatozoonosis in Shiraz, southern Iran. In addition, hematological alterations induced by natural hepatic zoonotic infectious were also evaluated.

## MATERIALS AND METHODS

**Animal rights statement: **This study was conducted under the supervision of the Iranian Society for the Prevention of Animal Cruelty and the Research Council of Shiraz University (IACUC No. 6387/63).

**Blood sampling and complete blood count analysis:** From May 2020 to December 2021, 108 blood samples were obtained from different dogs referred to the small animal department of the School of Veterinary Medicine of Shiraz University from different locations throughout south Iran. Dog demographic data, including age, gender, and comorbid disease, were extracted. Blood samples were taken from the cephalic vein of dogs in EDTA-coated tubes and kept at -20 °C until DNA extraction. Additionally, the hematological parameters consisted of hematocrit (HCT), Mean Corpuscular Volume (MCV), Red Blood Cell (RBC) count, Hemoglobin Concentration (Hgb), Mean Corpuscular Hemoglobin Concentration (MCHC), Mean Corpuscular Hemoglobin (MCH), total White Blood Cell (WBC) counts, and total Platelet (PLT) counts were assessed using a digital cell counter (Exigo, Stockholm, Sweden).

**DNA extraction and PCR amplification:** DNA was extracted by the DNA Purification Kit (Parstous; Blood DNA Extraction Kit), according to manufacturer instructions. The polymerase chain reaction (PCR) assay was conducted; also, to spot Hepatozoon spp. infections, the 18S rRNA gene was amplified. A reverse primer (5`-ACA ATA AAG TAA AAA ACA YTT CAA AG-3) and a forward primer (5`-GGT AAT TCT AGA GCT AAT ACA TGA GC-3`) amplified a 593 bp fragment. The PCR cycles started at 95°C for 7 min; then, 35 cycles of denaturation for 60 sec at 94°C, primers annealing for 60 sec at 60°C, and extension for 45 sec at 72°C were performed. The final extension took only one step for 5 min at 72°C. Gel electrophoresis of 1.5% Red safe (Intron Biotechnology, Korea)-stained agarose gel was conducted at 100 volts for 45 min. After gel electrophoresis, PCR products were visualized and photographed on a transilluminator to detect positive bands at a 593 bp position alongside a 100 bp molecular weight marker. The PCR was performed in a final volume of 20 μl of reaction mixture containing 10 μl of PCR Master Mix (Amplicon, Denmark), 0.7 μl of each primer (7 pmol), 6.6 μl of nuclease-free water, and 2 μl of DNA template (~20 ng). 

**DNA sequencing: **The multiple sequence alignment was done by BioEdit Software using the CLUSTAL W method. The representative nucleotide sequence of the Iranian Hepotozoon spp. obtained once sequencing was compared to its other reported sequences in the database of the NCBI utilizing the Basic Local Alignment Search Tool (BLAST) http://blast.ncbi.nlm.nih. gov/Blast.cgi. A phylogenetic tree was created by MEGA X software utilizing the bootstrap phylogeny testing at 1000 replications by the Maximum Composite likelihood method [8].

**Statistical Analysis:** Obtained data were entered into the Statistical Package for the Social Sciences (SPSS) (version 24.0). The Mann-Whitney U test was used in a non-parametric statistical analysis to evaluate if there were statistically significant differences in the tested blood parameters for PCR positive and negative cases. The student t-test or non-parametric statistical (Mann-Whitney U test) analysis was employed to determine any statistically significant differences between the hematological parameters assessed for PCR positive and negative cases (i.e., RBC, HCT, Hb, MCV, MCH, MCHC, WBC, and Plt). In addition, Chi-square and Fisher's exact tests were used to assess the relationship between risk factors and parasite infection. A p-value below "0.05" was considered significant. 

## RESULTS

**Figure 1:**
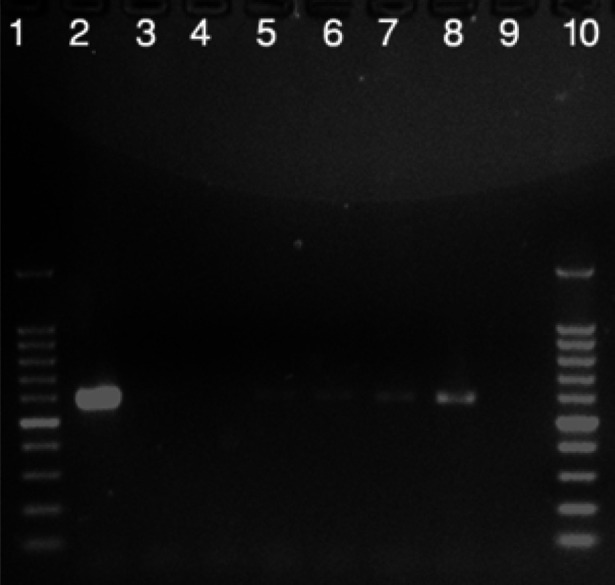
Electrophoresis of PCR products of *Hepatozoon* *canis* in agarose gel stained with Red Safe; Positive samples (lanes 5 to 8), negative samples (lanes 3 and 4), positive controls (lane 2), and negative controls (9) as compared with DNA molecular weight marker (Gene ruler 100 bp DNA Ladder; Fermentas Life Sciences, Carlsbad, CA, USA) (lanes 1 and 10).

Eight blood samples were positive (7.40%, 95% CI: 3.25-14.07%) in PCR assay for Hepatozoon spp. and produced 593 bp fragments. In contrast, a microscopic examination of the blood smears showed that out of 108, only one (0.93%) dog was found positive for Hepatozoon spp. gamonts within neutrophils. No evidence of clinical signs attributable to infection was detected in positive dogs. All obtained sequences of Iranian Hepatozoon sp (GenBank accession nos.: MZ708741- MZ708744) had 100% similarity to each other and showed 98.47-100% similarity with other reported *H. canis* species sequences from the NCBI database by BLAST. However, the sequence of Iranian Hepatozoon sp. was different from *H. americanum* sequences based on the GenBank data (Fig. 1). 

**Figure 2:**
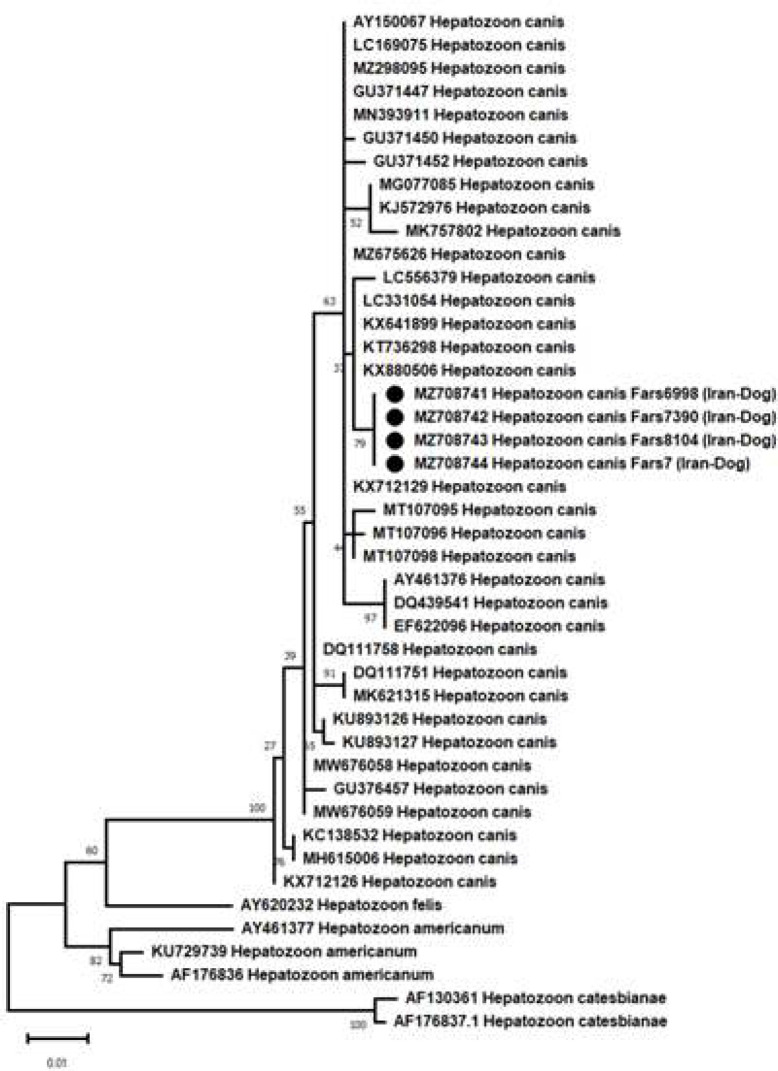
Phylogenetic analysis of 18S rDNA gene sequences of the *Hepatozoon* *canis* strain in this study compared with other related strains in the NCBI GenBank; The circle shape (●) indicates Iranian *Hepatozoon* *canis* strains; The scale bar shows nucleotide substitutions; *Hepatozoon* *catesbianae* strains (AF130361 and AF176873) are used as out-group; Bootstrap percentages are provided at the tree nodes.

**Figure 3:**
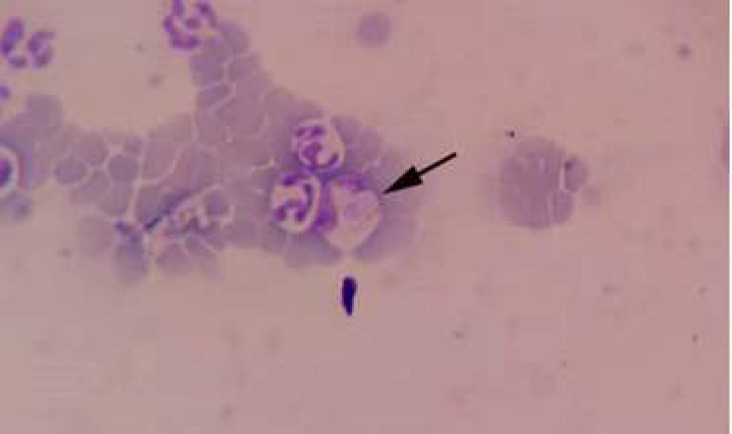
Blood film shows gamont of *Hepatozoon canis* stained in the cytoplasm of the circulating neutrophils of the infected dog.

The phylogenetic analysis used the partial sequences of the 18S rRNA gene from four dogs. The sequences were highly similar to those of *H. canis* isolates from Africa (LC556379), Turkey (KX641899), Ardabil (KT736298), and Tehran (KX880506). The phylogenetic tree also showed the Iranian Hepatozoon sp. positioned into a different major group compared to the H. americanum taxa (Fig. 2 and 3).

The hematological analysis revealed that PCR-positive dogs had a non-significant decrease in HCT, Hb, RBC, MCV, MCH, and Plt values compared to the non-infected control group. However, the infected dogs showed a significant (p<0.05) rise in total lymphocyte and neutrophil counts in the blood samples (Table 1). 

The risk factors evaluation, the signalment parameters, including genus, breed, ages (above 2 years compared to less than two years), and different seasons of the sampling had no significant differences between *H. canis*-infected and -non-infected dogs in this study (p>0.05).

**Table 1:** Comparison of hematological values (mean and standard error in relation to different RBC indices) of PCR-positive and -negative dogs

**Parameter ** **(Unit)**	**PCR positive (n=8)** **Mean ± SE**	**PCR Negative** **Mean ± SE**	**p-value**
Hct (%)	39.77 ± 4.11	45.20 ± 1.07	0.100
Hb (g/dl)	13.72 ± 1.63	15.85 ± 0.42	0.102
RBC (10^6^/µl)	5.72 ± 0.56	6.05 ± 0.14	0.430
WBC (10^3^/µl)	12.67 ± 2.08	10.08 ± 0.47	0.083
Neut (10^3^/µl)	10.99 ± 1.23^a^	7.27 ± 0.39^b^	0.003
Lymph (10^3^/µl)	2.53 ± 0.27^a^	1.76 ± 0.09^b^	0.012
Mono (10^3^/µl)	0.18 ± 0.08	0.38 ± 0.05	0.192
Eosin (10^3^/µl)	0.09 ± 0.05	0.2 ± 0.03	0.230
Band Neut (10^3^/µl)	0.54 ± 0.13	0.40 ± 0.05	0.411
MCV (fl)	70.41 ± 4.57	75.00 ± 0.99	0.148
MCH (pg)	24.25 ± 1.86	26.29 ± 0.43	0.137
MCHC (g/dl)	34.2 ± 0.92	34.94 ± 0.23	0.296
RDW (%)	16.2 ± 0.86	15.58 ± 0.23	0.372
MPV (fl)	7.81 ± 0.26	8.08 ± 0.17	0.607
Plt (10^3^/µl)	258.12 ± 54.64	344.37 ± 19.74	0.137

Hematological parameters included hematocrit (Hct), hemoglobin (Hb), red blood cell (RBC), mean cell volume (MCV), mean cell hemoglobin concentration (MCHC), mean cell hemoglobin (MCH), and platelet (Plt); Statistically significant factors (P<0.05) are shown with different superscripted letters. Different superscript letters indicate significant differences in each row.

## DISCUSSION

*H. canis* has a higher geographical distribution (Europe, Asia, Africa, and America) compared to *H. americanum*, which is limited to America. In the current research, 8 samples were detected positive (7.4%) by molecular analysis. While microscopically examining thin blood smears detected gamonts in neutrophils only in one sample. Hepatozoon infections have been spotted by blood smear examination ranging from 1% in Iran [9] to 39.2% in Brazil [10] and 10% in Cuba [3]. Hepatozoonosis in blood has been detected by the PCR method in Cuba (47.5%), the Czech Republic (50%), Brazil (53.3%), Japan (42.9%), Italy (57.8%), Pakistan (11.9%), Thailand(11.4%), Kyrgyzstan (28.8% ), and Turkey (44.67% ) [11].

Canine hepatozoonosis has been reported in some regions in Iran. According to a study from the Northeast of Iran, the prevalence of *H. canis* detected by PCR was about 8% [12]. In 2012, another study investigated Hepatozoon infections in dogs in Mashhad by cytological examination [9]. Similarly, Khoshnegah et al. demonstrated the Hepatozoon canis gametocytes in peripheral blood and bone marrow smear in an 11-year-old male dog for the first time in Iran [4]. Soltani et al. detected Hepatozoon infections in stray dogs by the PCR method in Tehran. The prevalence of *H. canis* was reported at about 22% [13].

 The current statistical data analysis shows a poor agreement between the microscopic examination and the PCR results for H. canis. Similarly, Rucksak et al. argue that microscopic detection of hepatozoonosis has a 36.73% false negative result compared to the PCR results [14].

Globally, the prevalence of Canine Hepatozoonosis is affected by several factors, such as the geographical region, vector distribution, climate, tick infestations, seasonal sampling, dog population, and the methods used to assess the infection [15]. According to some published studies, hepatozoonosis infection peaks during the summer. This relationship may partly be explained by the presence of ticks in this season [16].

Some previous studies point out the notable paucity of cytologic examination in the diagnosis of canine hepatozoonosis. Bhusri et al. and Sasanelli et al. showed that identifying canine hepatozoonosis by the microscopic examination method had low sensitivity and that using the PCR technique and 18S rRNA amplification was the well-established method in the detection of canine hepatozoonosis [17-20]. Like Mylonakis et al., the current research revealed that the statistical analysis of the signalment parameters, the origin of living, and different year seasons had insignificant differences between H. canis-infected and -non-infected dogs [21]. Contrary to the studies mentioned above, Baneth and Weigler showed rural dogs to be more infected with hepatozoonosis compared to urban ones [22].

Several pieces of research have challenged the effect of canine hepatozoonosis on the hematologic parameters of dogs. Several studies have explored neutrophilia and leukocytosis. Lea Vojta et al. showed eosinophilia, leukocytosis, and neutrophilia in 64.3%, 35.7%, and 21.4% of dogs with hepatozoonosis, respectively [23]. A retrospective study of hepatozoonosis demonstrated leukocytosis and neutrophilia in dogs [24].

In the current study, neutrophilia and lymphocytosis were detected in positive dogs. These outcomes are contrary to those of Adrian et al. (2021), who found leukopenia and neutropenia in infected dogs [3]. Sukullaya et al. showed that canine hepatozoonosis did not impact the hematologic parameters of dogs [25].

Consistent with the other studies, the hematological analysis revealed that PCR-positive dogs had lower HCT, Hb, RBC, MCV, MCH, and Plt than the non-infected control group. Adrina et al. focused on anemia as a hematologic result of hepatozoonosis in stray dogs. Other literature broadly supports this point that one of the complications of canine hepatozoonosis is anemia in dogs, followed by thrombocytopenia [26]. No evidence of clinical signs was detected in infected dogs in the present study. Likewise, Benjaporn et al. asserted the clinical sign of canine hepatozoonosis to be mostly asymptomatic unless under certain circumstances such as stress, immunosuppression, and coinfections diseases [17]. The investigation of canine hepatozoonosis in Argentina showed no clinical signs during the one-year follow-up period [27]. Conversely, according to Gondim et al. (1998) and Khoshnegah et al., canine hepatozoonosis is categorized by clinical signs, including anorexia, weight loss, pale mucous membranes, diarrhea, vomiting, pain, and fever. Some clinical observations support this evidence that the most frequent clinical signs of hepatozoonosis are emaciation, anemia, and fever. Based on the current research findings, the prevalence of hepatozoonosis in dogs of southern Iran was 7.4%. The canine Hepatozoon species was isolated from *H. canis* according to sequence alignments. Also, *H. canis* was identified as the main causative agent for canine hepatozoonosis in this region. Nonetheless, more research on canine hepatozoonosis in other parts of Iran is necessary to understand the disease better.

## Conflict of Interest:

The authors declare that they have no conflicts of interest.
